# Endoplasmic reticulum stress is induced in the human placenta during labour

**DOI:** 10.1016/j.placenta.2014.11.005

**Published:** 2015-01

**Authors:** J.H.W. Veerbeek, M.C. Tissot Van Patot, G.J. Burton, H.W. Yung

**Affiliations:** a Centre for Trophoblast Research, Department of Physiology, Development, and Neuroscience, University of Cambridge, UK; b University Medical Center Utrecht, Division of Perinatology, Department of Obstetrics, The Netherlands

**Keywords:** Endoplasmic reticulum stress, Labour, Placenta

## Abstract

Placental endoplasmic reticulum (ER) stress has been postulated in the pathophysiology of pre-eclampsia (PE) and intrauterine growth restriction (IUGR), but its activation remains elusive. Oxidative stress induced by ischaemia/hypoxia-reoxygenation activates ER stress *in vitro*. Here, we explored whether exposure to labour represents an *in vivo* model for the study of acute placental ER stress. ER stress markers, GRP78, P-eIF2α and XBP-1, were significantly higher in laboured placentas than in Caesarean-delivered controls localised mainly in the syncytiotrophoblast. The similarities to changes observed in PE/IUGR placentas suggest exposure to labour can be used to investigate induction of ER stress in pathological placentas.

## Highlights

• The process of labour activates endoplasmic reticulum stress response pathways in the human placenta.
• Labour may be used as an *in vivo* model to investigate molecular changes induced by endoplasmic reticulum stress.
• Downstream consequences of endoplasmic reticulum stress may compromise placental function in laboured samples *ex vivo*.

## 1 Introduction

Insufficient remodelling of spiral arteries is thought to underlie several pregnancy complications, such as pre-eclampsia (PE), intrauterine growth restriction (IUGR) and preterm labour [1], [2], [3]. Secondary to this maldevelopment, placental malperfusion may result in a repetitive hypoxia-reperfusion type of injury resulting in oxidative stress, which can compromise normal function of cellular components including mitochondria and the endoplasmic reticulum (ER) [4], [5].

The ER is the organelle responsible for the synthesis and post-translational modification of secretory and membrane proteins, prior to delivery to the Golgi apparatus for final targeting. The ER has its own intricate network of signalling proteins that continually senses and communicates ER status to the cell. When ER homeostasis is perturbed, these proteins coordinate the Unfolded Protein Response (UPR), a signalling cascade that aims to restore ER homeostasis and relieve the stress or induce apoptosis if this process fails [6].

Three highly conserved signalling pathways are activated in the UPR, including PRKR-like endoplasmic reticulum kinase (PERK) which in turn phosphorylates eukaryotic initiation factor 2 subunit α (eIF2α) and inhibits non-essential protein synthesis; activating transcription factor 6 (ATF6) which up-regulates ER chaperones (GRP78 and GRP94) to increase folding capacity; and inositol requiring protein 1 (IRE1) which in turn activates X-box binding protein 1 (XBP-1) and TRAF2 for up-regulating phospholipid biosynthesis, promoting mis-folded protein degradation and provoking inflammatory response [6], [7]. These three signalling pathways are usually activated in a sequential manner dependent on the severity of ER stress stimulation [8], [9].

We recently showed that ER stress might contribute to the pathophysiology of early-onset PE and IUGR, but not late-onset PE [10]. Our laboratory has previously demonstrated that exposure of placentas to labour can provide a useful *in vivo* model for studying cellular changes induced by oxidative stress seen in the pre-eclamptic placenta. In this study we further tested whether ER stress can also be induced by the labouring process in placentas from healthy pregnancies and the potential use a *in vivo* model to study placental cellular changes to ER stress in the absence of maternal factors.

## 2 Methods

A total of 16 placental samples were used for the study, including 8 caesarean section controls and 8 labour placentas. All placentas were delivered at term by standard vaginal delivery or by elective non-labouring caesarean section (CS) from normotensive healthy singleton pregnancies. Both groups had no history of cigarette smoking, diabetes, autoimmune diseases, thrombophilic conditions or complicated pregnancies.

Detailed description of sample collection was published previously [11]. Briefly, samples were collected in the University College Hospital in London where local ethics committee approved of the study and all patients signed informed consent. For each placenta, small pieces of tissue from separate lobules were randomly taken and rinsed in PBS to remove excess blood, blotted dry and snap-frozen in liquid nitrogen within 10 min of delivery; the samples were stored at −80 °C.

For immunohistochemistry, one full-thickness section placed into 10% buffered formalin for 12–24 h before embedding in paraffin wax according to standard procedures. Sections were cut at 6 μm thickness. After dewaxing and blocking of endogenous peroxidases, the sections were incubated with non-immune serum for 1 h. Rabbit polyclonal to GRP78/BiP (ab21685, Abcam, Cambridge UK) was diluted 1:3000 and incubated overnight at 4 °C. Vectastain Elite ABC kits (Vector Laboratories, Burlingame, CA) and Sigma*Fast* diaminobenzidine (Sigma–Aldrich) were used to detect binding. Sections were lightly counterstained with haematoxylin. GRP78 intensity was scored in a semiquantitative fashion scoring the intensity of syncytial staining in 10 random fields at 25× magnification. The staining intensity was graded as 0 (no staining), 1+ (weak), 2+ (moderate), or 3+ (strong). The H-score was calculated using the following formula; H-Score = (% at 0) * 0 + (% at 1+) * 1 + (% at 2+) * 2 + (% at 3+) * 3.

Western blotting was performed as described elsewhere [9].

Statistical analyses were performed using the Prism GraphPad verson 6.0. Differences between study groups were analysed by the two-tailed Mann–Whitney *U* test and plotted as Box & whiskers with 10–90 percentile. We considered differences to be statistically significant at *p* < 0.05.

## 3 Results

Average duration of labour was 12.3 h (SD ± 3.8) and caesarean controls (CS) were free from labour. Mean placental weight (604 g vs. 571 g; *p* = 0.43) and birth weight (3489 g vs. 3270 g; *p* = 0.59) were not statistically different between the two groups.

**Fig. 1:**
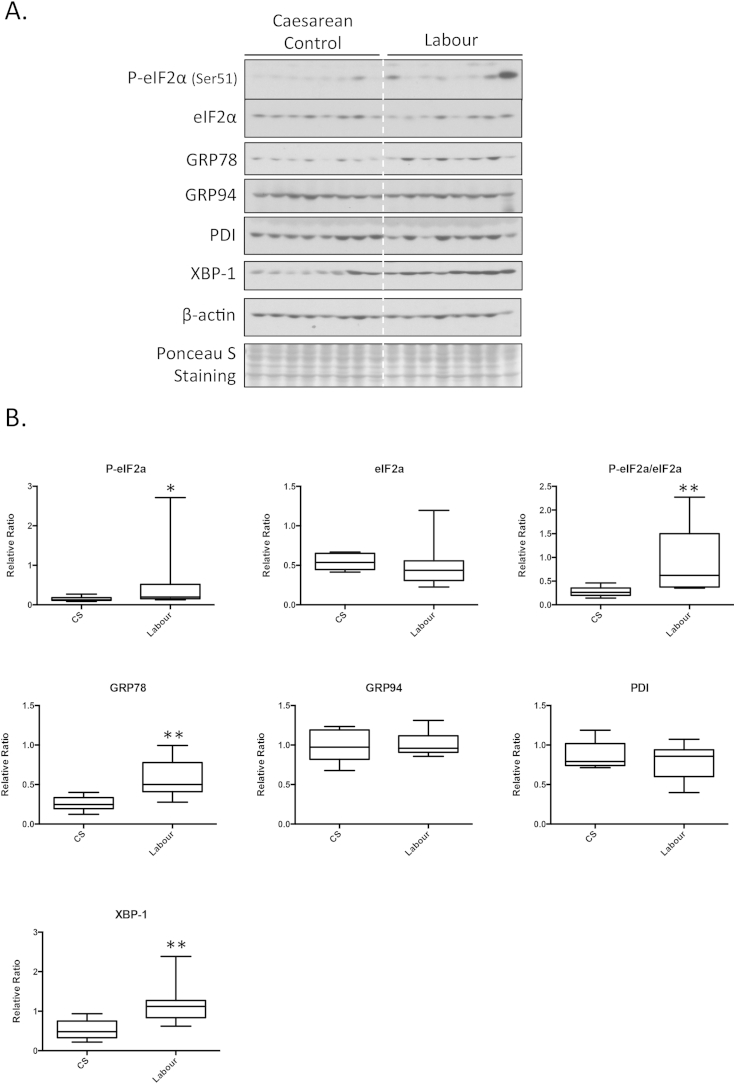

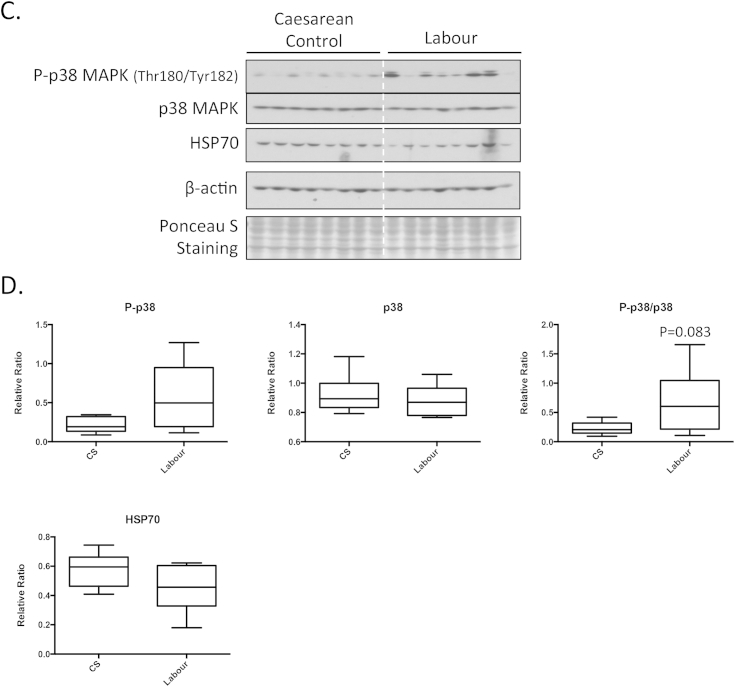
Increase of ER stress markers in the vaginal delivery labour placentas compared to caesarean section placentas. A) Equal amount of proteins were subjected for Western blotting analysis with antibodies specific against a number of ER stress markers. β-actin was used for the loading control. B) Densitometry of bands expressed relative to normal controls (100%). Phosphorylation status is presented as the ratio between phosphorylated and total protein, both normalized to β-actin. Data are mean ± SD for eight placentas per group. “*” and “**” indicate *p* < *0.05* and *p* < *0.01* respectively.

Western blotting showed significantly higher protein levels of GRP78, XBP-1 and p-eIF2α (Fig. 1A and B) in placentas exposed to labour compared to CS controls. A trend (*p* = 0.083) was seen in the ratio of P-p38 to p38, which was higher in the laboured group (Fig. 1C and D).

**Fig. 2:**
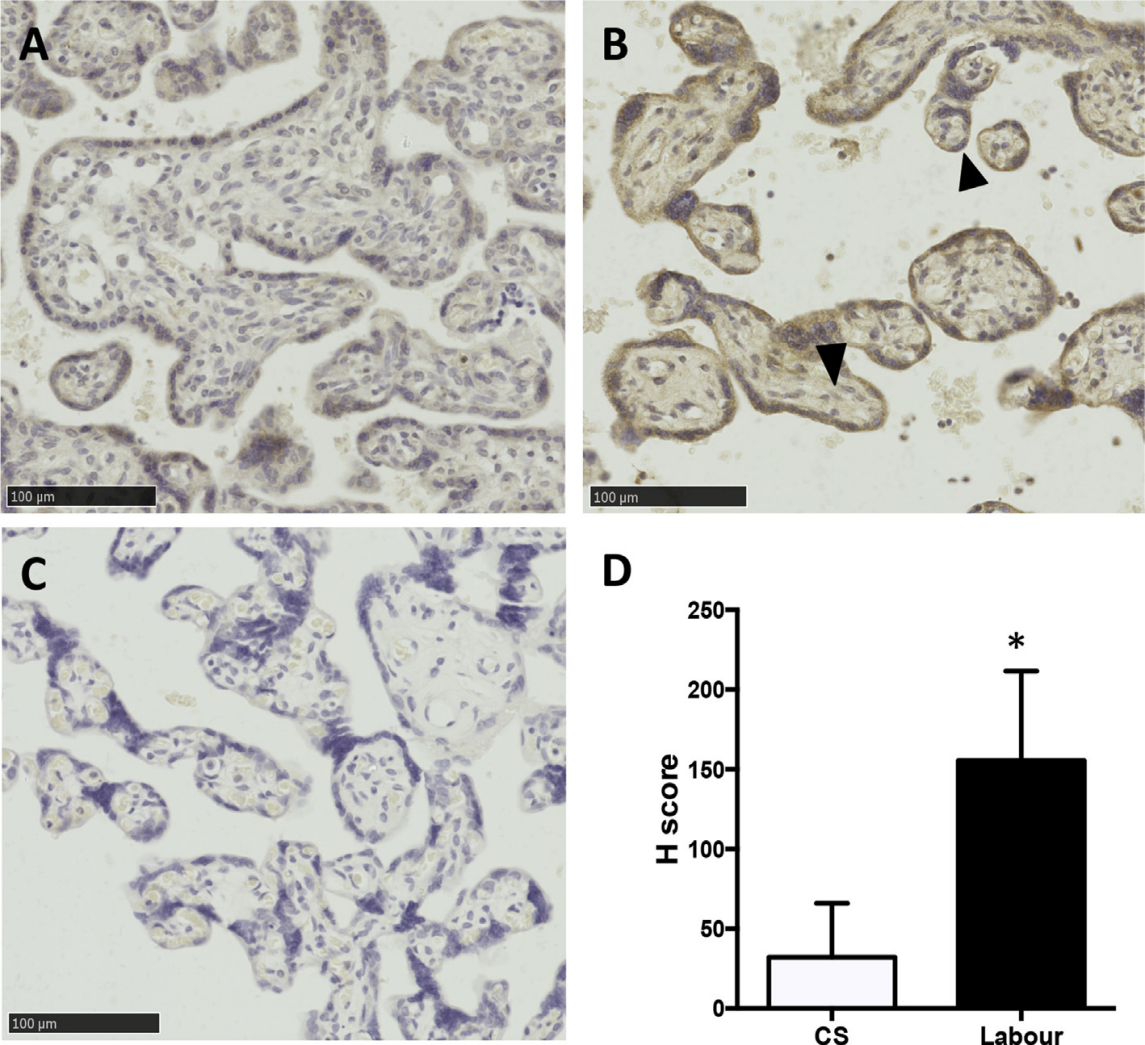
Higher GRP78 immunoreactivity in laboured placenta. A) Caesarean control; B) Labour; C) No primary antibody negative control; D) Semi-quantitation of GRP78 immunoreactivity in placentas using H-score. Data presented as mean ± SD, *n* = 8. * indicates *p* < 0.001. Arrowhead shows positive staining of GRP78 in syncytium. Scale bar = 100 μm.

Immunohistochemistry showed strong reactivity for GRP78 in laboured placentas, but not in the CS controls. Quantitation of immunoreactivity of GRP78 by H-score indicated an approximate 3 fold increased in its intensity (Fig. 2D). The majority of the positive staining was found in the syncytiotrophoblast while no or only weak staining was observed in stromal and endothelial cells (Fig. 2 A and B).

## 4 Discussion

In this study we provide evidence that placental ER stress can be induced by the process of labour, as indicated by the elevated levels of ER stress markers, including GRP78, XBP1 and P-eIF2α. The majority of ER stress induced by labour was found in the syncytiotrophoblast, consistent with the localization of ER stress observed in placentas from cases of IUGR and early-onset PE [9].

Placental ER stress has been implicated in many pregnancy disorders, including small-for-gestational age at high altitude, IUGR, early-onset PE and gestational diabetes, but not in late-onset PE [ [9], [12] and HWY, unpublished data]. ER stress response pathways can be induced by a variety of stimuli, including viral infection, hypoxia, oxidative stress and nutrient deprivation. We have previously demonstrated that oxidative stress induced by ischaemia/hypoxia-reoxygenation is a strong inducer of ER stress *in vitro* in human choriocarcinoma cells [8], [9]. Although the exact mechanisms are unclear, there are at least two potential pathways by which the oxidative and ER stress may be linked. Firstly, reduction of intracellular ATP levels during oxidative stress could inhibit SERCA channel activity in the ER membranes, resulting in perturbation of Ca^2+^ homeostasis and loss of function of the protein folding (PDI) enzymes. Secondly, formation of disulfide bonds during folding is an oxidative process and the PDI enzymes require molecular oxygen as an electron acceptor. A short electron transport chain is present within the ER, and, as in mitochondria, leakage can occur generating reactive oxygen species (ROS). Under normal conditions, approximately 25% of ROS generated in a cell arise within the ER, and this will be increased with attempts to refold misfolded proteins. Activation of PERK increases the synthesis of glutathione and also promotes the nuclear translocation of Nrf2, regulating antioxidant gene expression. Nonetheless, if these responses are overwhelmed, a feedback loop that further enhances ER stress will be formed.

Exposure to labour has been demonstrated to be a useful tool to study placental responses to oxidative stress induced by a physiological insult *in vivo* [13]. Our results indicate that it may also be used to study acute responses in ER stress signalling pathways. Placental oxidative and ER stress have been implicated in the pathophysiology of early-onset PE. However, elucidating their role in this syndrome is confounded by the presence of the maternal pro-inflammatory milieu, making it difficult to distinguish between cause and effect. Exposure to labour may therefore present the opportunity to study placental cellular changes to oxidative and ER stresses in the absence of maternal factors.

An alternative explanation is that placental stresses induce labour. We have previously reported that induction of oxidative stress in the mouse placenta is associated with upregulation of cyclooxygenase enzymes, and proposed this possibility [14]. ER stress response pathways can also cause increase secretion of pro-inflammatory cytokines [7] that are known to promote labour. Separating cause and effect is impossible in the human placenta, and further studies in animal models are required.

Our results further suggest that caution should be taken when interpreting gene expression or other data obtained from placentas exposed to labour, for they may be subjected to stress-induced protein synthesis inhibition and other downstream consequences of ER stress.

## Disclosure statement

The authors report no conflict of interest.
